# Supplementary material for: A Global Screen for Assembly State Changes of the Mitotic Proteome by SEC-SWATH-MS
Source: Cell Syst. 2020 Feb 26;10(2):133–155.e6. doi: 10.1016/j.cels.2020.01.001 (PMC7042714; doi:10.1016/j.cels.2020.01.001)

Q8TAE8 | G45IP_HUMAN | GADD45GIP1 MRPL59 PLINP1 PRG6
Monomer MW [kDa]: 25.384 Monomer expected elution fraction: 52

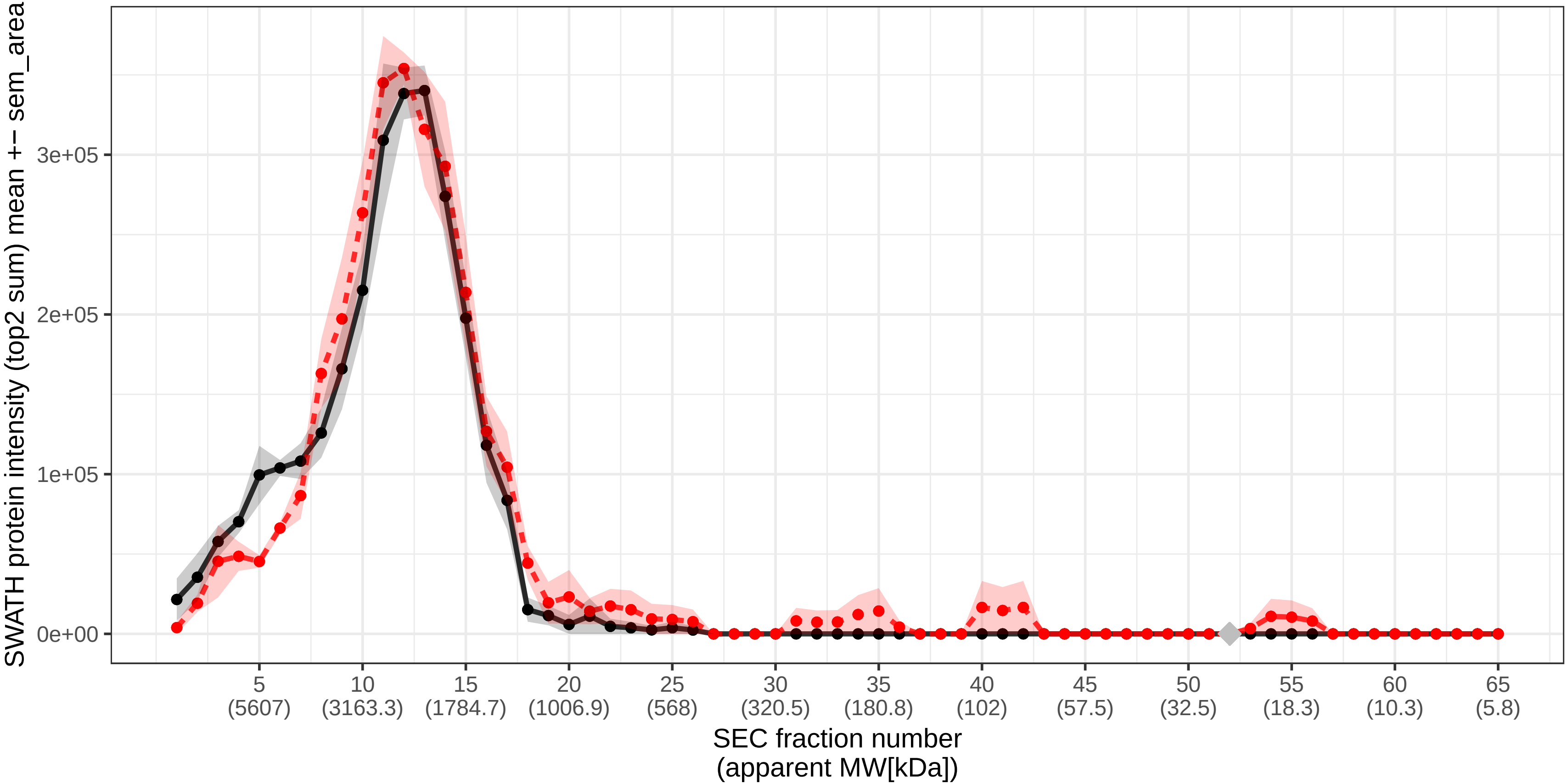

Supplement: Data S1. SEC-SWATH-MS Protein Chromatograms, Related to Figure 1 [file mmc6.zip › SECchrom_Q8TAE8_G45IP_HUMAN_GADD45GIP1_MRPL59_PLINP1_PRG6.pdf]
